# A Design of Analog Front-End with DBPSK Demodulator for Magnetic Field Wireless Network Sensors

**DOI:** 10.3390/s22197217

**Published:** 2022-09-23

**Authors:** S. Ali Hosseini Asl, Behnam S. Rikan, Arash Hejazi, YoungGun Pu, Hyungki Huh, Yeonjae Jung, Keum Cheol Hwang, Youngoo Yang, Kang-Yoon Lee

**Affiliations:** 1 Department of Electrical and Computer Engineering, Sungkyunkwan University, Suwon 16419, Korea; 2 SKAIChips Co., Ltd., Suwon 16419, Korea

**Keywords:** DBPSK, demodulator, AFE, magnetic field communication, wireless network sensors

## Abstract

This paper presents an on-chip fully integrated analog front-end (AFE) with a non-coherent digital binary phase-shift keying (DBPSK) demodulator suitable for short-range magnetic field wireless communication applications. The proposed non-coherent DBPSK demodulator is designed based on using comparators to digitize the received differential analog BPSK signal. The DBPSK demodulator does not need any phase-lock loop (PLL) to detect the data and recover the clock. Moreover, the proposed demodulator provides the detected data and the recovered clock simultaneously. Even though previous studies have offered the basic structure of the AFEs, this work tries to amplify and generate the required differential BPSK signal without missing data and clock throughout the AFE, while a low voltage level signal is received at the input of the AFE. A DC-offset cancellation (DCOC), a cascaded variable gain amplifier (VGA), and a single-to-differential (STOD) converter are employed to construct the implemented AFE. The simulation results indicate that the AFE provides a dynamic range of 0 dB to 40 dB power gain with 2 dB resolution. Measurement results show the minimum detectable voltage at the input of AFE is obtained at 20 mV peak-to-peak. The AFE and the proposed DBSPK demodulator are analyzed and fabricated in a 130 nm Bipolar-CMOS-DMOS (BCD) technology to recover the maximum data rate of 32 kbps where the carrier frequency is 128 kHz. The implemented DCOC, cascaded VGA, STOD, and the demodulator occupy 0.15 ${\mathrm{mm}}^{2}$, 0.063 ${\mathrm{mm}}^{2}$, 0.045 ${\mathrm{mm}}^{2}$, and 0.03 ${\mathrm{mm}}^{2}$ of area, respectively. The AFE and the demodulator consume 2.9 mA and 0.15 mA of current from an external 5 V power supply, respectively.

## 1. Introduction

The development of wireless communication devices to monitor difficult-to-access areas (e.g., when a wireless sensor is used underground or underwater to monitor the fluency of oil, gas, etc.) has increased. The challenges of power consumption, noise, gain, linearity, and high-cost fabrication in radio frequency integrated circuit (RF IC) design led to the design of magnetic field wireless sensor networks (WSNs) as one of the alternative applications in short-range wireless communications [1,2,3].

Amplitude-shift keying (ASK) [4], on-off keying (OOK) [5], and phase-shift keying (PSK) [6] are some of the conventional data modulation techniques for wireless data transfer. In an ASK modulation technique, the data are carried by the amplitude. Therefore, the sensitivity of carrier amplitude to noise causes missing data in short-range applications when the input amplitude is very low. In other words, while the distance between transmitter (TX) and receiver (RX) is not very short, due to the attenuation of signal and sensitivity of carrier amplitude to noise, data would be missed. Hence, the ASK modulation technique is not recommended in this application.

To detect the data in a conventional coherent binary phase-shift keying (BPSK) demodulator, the clock first needs to be recovered to resample the BPSK signal [7]. In general, Costas loops, squaring loops, and demodulators are employed to adopt phase-lock loops (PLLs) to recover the required clock [8]. Hence, a considerable silicon die and power consumption have to be devoted to the PLL. Moreover, PLLs need a significant settling time to generate the operational clock. The proposed non-coherent DBPSK demodulator is designed based on using comparators and delay cells to digitalize input signals. In the proposed structure, there is no implemented PLL to detect data and recover clock. Furthermore, the detected data and recovered clock are generated at the same time.

Since the received analog BPSK signal at the input of the demodulator might not be large enough to guarantee the operation of the comparators (e.g., in the case of using the sensors underground or underwater), the received analog BPSK signal has to be amplified without any change in the shape of the signal. Moreover, in the proposed DBPSK demodulator, to detect the data and recover the clock, the inverted analog BPSK signal is required. Consequently, employing an analog front-end (AFE) to amplify and provide the differential analog signal is inevitable. At the interface of the architecture to the analog BPSK signal, the DC voltage of the input signal is adjusted to half of the supply voltage (VDD/2) by a DC-offset cancellation (DCOC), which is located at the first stage of the AFE. A cascaded variable gain amplifier (VGA) offers 0 dB to 40 dB of power gain to amplify the input analog signal. This variation in power gain guarantees coverage of a wide dynamic range of input voltage levels. A single-to-differential (STOD) converter is placed at the last stage of the AFE to generate the required differential analog BPSK signal.

## 2. Overall Architecture and Building Blocks

**Figure 1:**
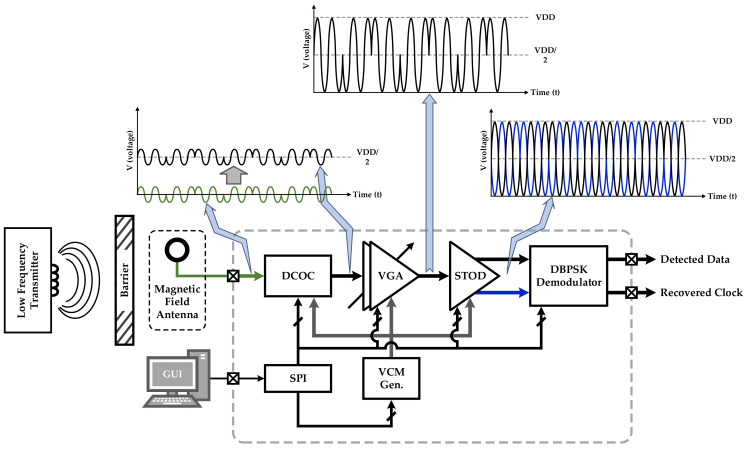
Architecture of the proposed RX.

Figure 1 illustrates the overall architecture of the proposed RX, which consists of a DCOC, a cascaded VGA, a STOD, a conventional common-mode voltage generator (VCM Gen.), and a serial peripheral interface (SPI).

In the proposed architecture, an external magnetic field antenna receives the transmitted analog BPSK signal from a low-frequency (LF) TX as stated in Figure 1. Depending on the distance and the barriers between the LF TX and the antenna, the attenuation of the transmitted analog BPSK signal cannot be constant. In other words, the amplitude of the received signal at the antenna is variable. Therefore, a reconfigurable gain amplifier is required to provide a dynamic range of amplification of the input signal from low to high voltage levels.

As indicated in Figure 1, the DCOC is employed at the first stage to adjust the DC voltage level of the input signal to the desired VCM (VDD/2) for the subsequence stage (VGA) where operational amplifiers (Op-amps) need to offer their best performance. The required VCM of 2.5 V (VDD/2) is provided by VCM Gen. To digitalize the input analog BPSK signal through the proposed DBPSK demodulator, the amplified input analog signal and its invert are required. The STOD circuit converts the single input analog signal to the required differential signal [9]. Through the SPI, a graphic user interface (GUI) programmer on a computer controls the RX’s digital controller.

## 3. Hysteresis Comparator Analysis

**Figure 2:**
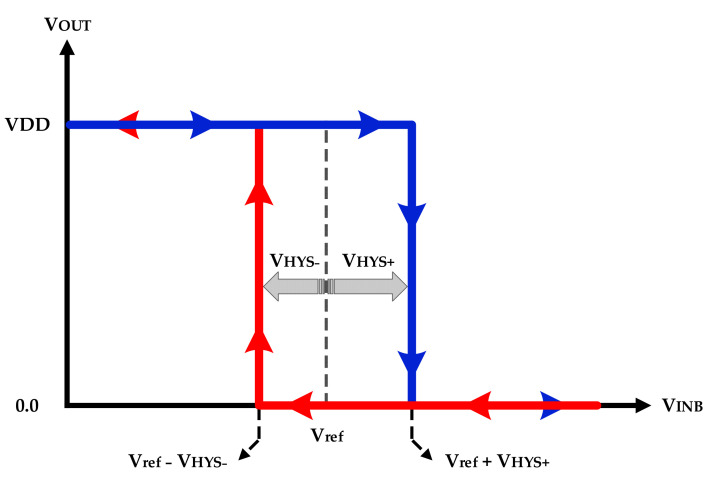
Loop characteristics of an inverting comparator.

Comparators are basic circuits to convert an analog signal to its digital format. To switch the output of a comparator from 0 to VDD and vice versa, a hysteresis comparator offers the possibility of having different down ($V_{HYS-}$) and up ($V_{HYS+}$) threshold voltages. The loop characteristics of an inverting comparator are illustrated in Figure 2.

The operation of the inverting comparator would be described in two scenarios: (1) an analog signal is applied to the negative input of the comparator, while the positive input is connected to a reference voltage ($V_{ref}$). In this case, the input signal is compared with $V_{ref}$, and the output shows the inverted digitalized signal. (2) A differential analog sinewave signal with a DC level: in this scenario, the negative input is compared with the DC level, which can be assumed as $V_{ref}$. The output voltage of the inverting comparator can be described by the following expression:

(1) $$\left\{\begin{array}{c} V_{INB}>V_{ref}-V_{HYS-};\;V_{OUT}=0\;\;\;\\ V_{INB}<V_{ref}+V_{HYS+};\;V_{OUT}=VDD \end{array}\right.\;$$

**Figure 3:**
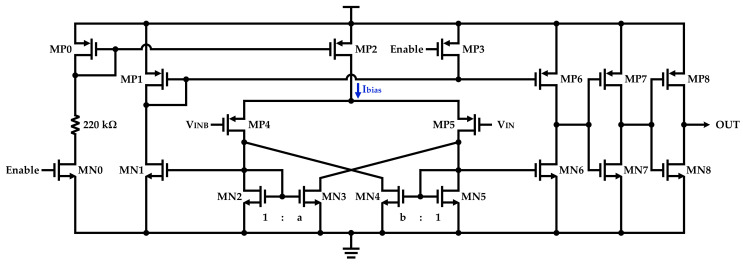
Structure of the implemented hysteresis comparator.

The structure of a hysteresis comparator is stated in Figure 3. In this structure, $V_{HYS-}$ and $V_{HYS+}$ can be defined by MN3 and MN4 [10]. $V_{HYS-}$ and $V_{HYS+}$ can be calculated by the following expressions [10]:

(2) VHYS−=2IbiasµCoxWL4,5×b−1b+1 

(3) VHYS+=2IbiasµCoxWL4,5×a−1a+1 

where a and b are greater than 1 and described by the *W* ratio of MN3 with MN2 and MN4 with MN5, respectively. It is worth mentioning that the output voltage of the hysteresis comparator is dependent on the values of the input voltage at the same time and its passed time.

## 4. DBPSK Demodulator

**Figure 4:**
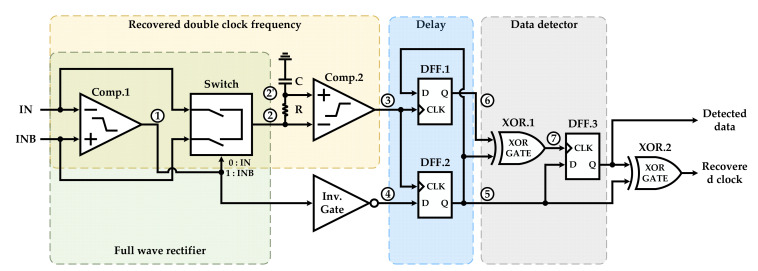
Structure of the proposed DBPSK demodulator.

**Figure 5:**
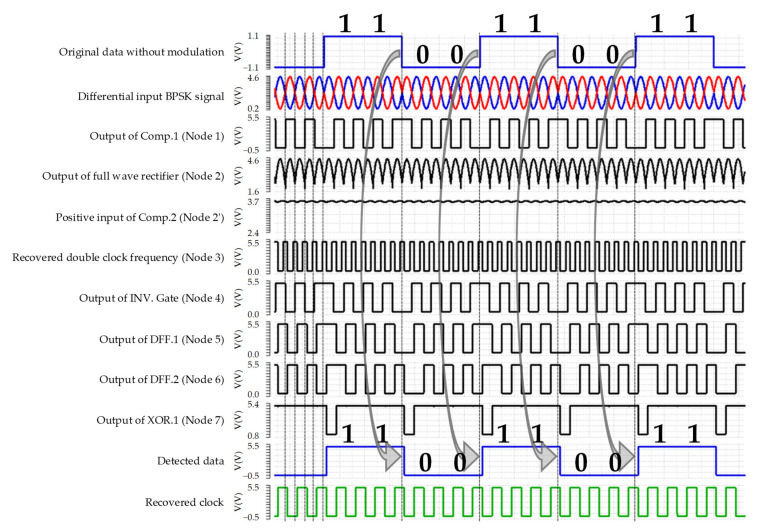
Simulated timing diagram of the proposed non-coherent DBPSK demodulator.

The structure of the proposed DBPSK demodulator is illustrated in Figure 4. Two hysteresis comparators, three dynamic flip-flips (DFFs), two XOR gates, and an inverter gate are formed as the building blocks of the proposed DBPSK demodulator to detect the data and recover the clock. Figure 5 depicts the simulation results of the proposed DBPSK demodulator. To detect the data and recover the clock, a double clock frequency from the differential BPSK signal is recovered by employing a full wave rectifier and a comparator (Comp.2).

The specified full-wave rectifier in Figure 4 consists of a comparator (Comp.1) and a switch that is controlled by the output of Comp.1. The operating principles of the proposed full-wave rectifier are as follows: when the input voltage level of the negative input (IN) is greater than ($VDD/2)+V_{HYS+}$, the output of Comp.1 (Node 1) is 0. In this case, the switch connects the IN to the negative input of Comp.2, while if the voltage level of the negative input of Comp.1 is lower than down ($VDD/2)-V_{HYS-}$, the output voltage of the comparator is VDD; hence, INB appears at the input of Comp.2 through the switch. Consequently, the output of the switch (Node 2) presents the full-wave rectified signal with DC level of VDD/2 as shown in Figure 3. The provided low-path filter (LPF) by (R = 1 MΩ) and (C = 20 pF) at the inputs of Comp.2 ensures the voltage difference of ΔV between the inputs of Comp.2. As the negative input swings, the Comp.2 recovers the double frequency clock with respect to crossing points (Node 3).

The implemented DBPSK demodulator using delay and a data detector monitors the location of rising and falling edges of the unmodulated data where the pulse width of the output of Comp.1 lasts longer, and some unmodulated edges are skipped. Since the double frequency clock is used to sample the BPSK signal to detect the data, the recovered clock and detected data are generated synchronously. To simulate the operation of the proposed DBSPK demodulator, the data signal is multiplied with a sinewave signal (carrier signal) to provide the required modulated signal, where the amplitude of the modulated BPSK signal is defined by the amplitude of the carrier signal. Figure 5 shows the original data signal without modulation, the differential modulated BPSK signal, the internal digitalized signals of the demodulation, the detected data, and the recovered clock.

## 5. Analog Front-End

**Figure 6:**
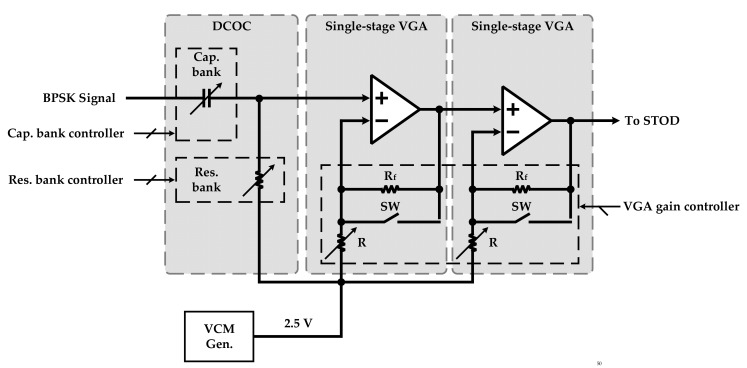
Block diagram of the DCOC and the cascaded VGA.

The DCOC circuit is located at the interface of the AFE to regulate the DC voltage level of the received analog BPSK signal to the desirable VCM of 2.5 V, which is generated by the conventional VCM Gen. As indicated in Figure 6, the DCOC Cap. bank (10–90 pF) blocks the DC level voltage of the input signal and adjusts the DC level voltage to 2.5 V through the DCOC Res. Bank (1–3.3 MΩ).

Two single-stage VGAs are implemented to construct the cascaded VGA block as depicted in Figure 6. The provided power gain of a single-stage VGA by using $R_{f}$ and *R* in the feedback is given by the following expression:

(4) $$\frac{V_{out}}{V_{in}}=1+\frac{R_{f}}{R}\;$$

Therefore, by using a reconfigurable resistor (*R*) in the feedback path, as indicated in Figure 6, a dynamic range of power gain from 0 dB to 20 dB with 2 dB resolution is obtained. It is noteworthy that 0 dB gain is achieved when the switch (SW) connects the output of the operational amplifier (Op-Amp) to its negative input; in this condition, the VGA operates as an analog buffer. Thus, the total dynamic range of amplification from 0 dB to 40 dB of power gain is provided by the cascaded VGA to amplify the received BPSK signal. It should be noted that the cutoff frequency of DCOC and the gain of cascaded VGA are controlled through the SPI part.

**Figure 7:**
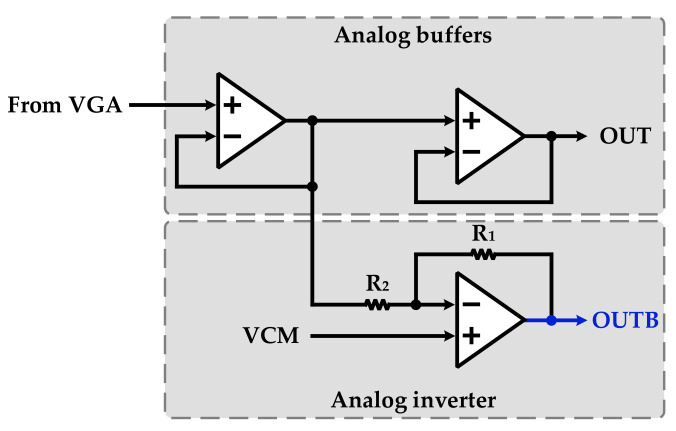
Block diagram of the STOD.

As discussed in the DBPSK demodulator section, to detect the data and recover the clock, the differential signal of the analog BPSK signal is required. The STOD consists of two analog buffers and an analog inverter to convert the single BPSK signal to its differential form as illustrated in Figure 7. The gain of the analog inverter can be written by following equation:

(5) $$\frac{V_{out}}{V_{in}}=-\frac{R_{1}}{R_{2}}$$

Since the gain of the analog invert should be −1, $R_{1}$ and $R_{2}$ are equal. It should be noted that the required VCM for operation of the analog inverter circuit is provided by VCM Gen.

## 6. Experimental Results

**Figure 8:**
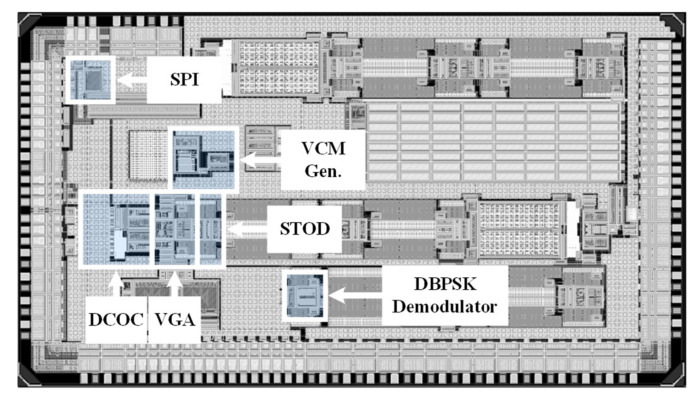
The top layout of the chip and location of AFE, DBPSK demodulator, SPI, and VCM Gen.

**Figure 9:**
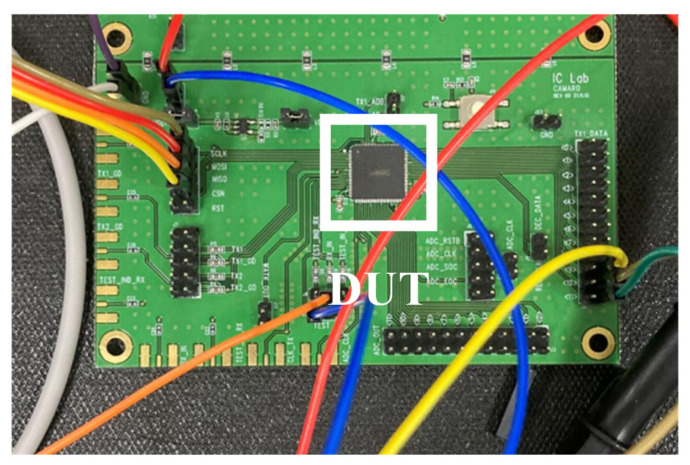
PCB and the device under test of the proposed architecture.

The proposed fully integrated AFE with the DBPSK demodulator for short-range magnetic field WSN is fabricated in a 130 nm Bipolar-CMOS-DMOS (BCD) technology with an active area of 0.67 ${\mathrm{mm}}^{2}$ and 0.03 ${\mathrm{mm}}^{2}$, respectively. The proposed DBPSK demodulator offers 25% data-rate-to-carrier frequency (DRCF). Figure 8 indicates the location of AFE, DBPSK demodulator, SPI, and VCM Gen. in the top layout. The print circuit board (PCB) and the device under test (DUT) for measurement is stated in Figure 9. The required supply voltage is offered by an external 5 V power supply to measure the performance of the AFE and the DBPSK demodulator. The modulated BPSK signal is provided by multiplying a sinewave (carrier signal) and a pulse (data signal).

**Figure 10:**
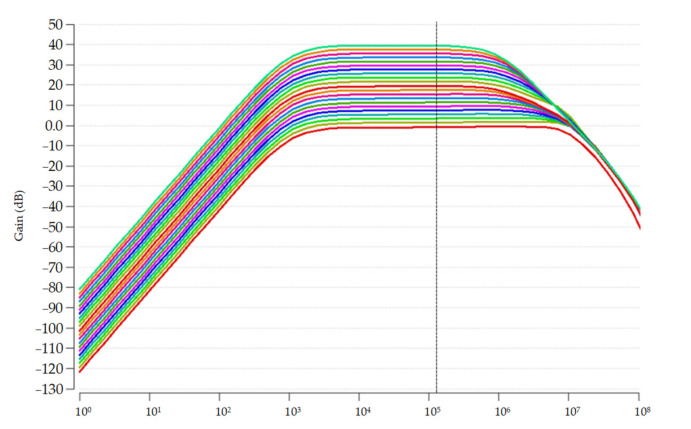
Post-layout simulation results of the provided power gain by AFE.

Figure 10 indicates the post-layout simulation results of the gain performance throughout the AFE, which is provided by the cascaded VGA. AFE can offer a wide range of power gain from 0 dB to 40 dB with 2 dB resolution.

**Figure 11:**
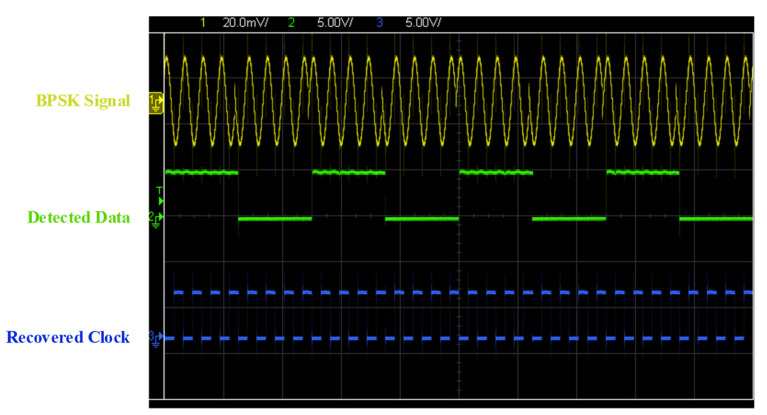
Measurement results of the device under test.

Figure 11 illustrates the measurement results of the detected data and recovered clock with 50% duty cycle. The measurement results show the received BPSK signal is successfully amplified by AFE and demodulated through the proposed DBPSK demodulator.

To compare the performance of the proposed DBPSK demodulator with previous structures, two figure of merits (FoMs) are suggested [11,12]. The maximum DRCF, power consumption, and occupied area are the most important factors to summarize the performance of a demodulator. Therefore, the suggested $FoM_{1}$ and $FoM_{2}$ can be written by following expressions [11,12]:

(6) $$FoM_{1}=\frac{DRCF}{Power\left(\mathrm{mW}\right)}\;$$

(7) $$FoM_{2}=\frac{FoM_{1}}{A\left({\mathrm{mm}}^{2}\right)}\;$$

The summarized performance of the proposed DBPSK demodulator and comparison with other studies are reported in Table 1. When compared to recent works, the proposed demodulator provides superior DRCF performance while consuming only 0.75 mW of power. Furthermore, Table 1 depicts the FoMs of the proposed DBPSK demodulator and other works for a fair comparison.

## 7. Conclusions

In this article, the fully integrated AFE along with a DPBSK demodulator are implemented in a 130 nm BCD process with a die size of 0.7 ${\mathrm{mm}}^{2}$. The power consumption of the proposed DBPSK demodulator is 0.75 mW to detect the data and recover the clock. Experimental results show the system offers a maximum data rate of 32 kbps where the carrier frequency is 128 kHz.

## Figures and Tables

**Table 1:** Performance of the proposed DBPSK demodulator and comparison with other studies.

Parameter	This Work	[13]	[14]	[15]	[16]	[17]	[18]
Year	2022	2013	2015	2016	2018	2019	2021
Modulation scheme	DBPSK	OOK-PM	BPSK	OOK/ASK	BPSK	FSK	OOK/BFSK/DBPSK
Tech. (nm)	130 BCD	350	130 CMOS	180	180	130	180
$\mathrm{Active}\;\mathrm{area}\;({\mathrm{mm}}^{2}$)	0.03	0.36 *	0.084	N.A	0.137	0.222	N.A
Power (mW)	0.75	<0.4	1.4	0.184	0.217	0.184	0.054/0.01
Carrier freq. (MHz)	0.128	1	21	1	13.56	405	433
Data rate (kbps)	32.0	25	1312.5	50.0	211	2500	200
DRCF (%)	25	2.5	6.25	5	1.55	0.617	0.046
$FoM_{1}$	**33.33**	6.25	4.46	27.17	7.17	3.35	0.85/4.6
$FoM_{2}$	**1111**	17.36	53.09	N.A	52.53	15.09	N.A

$FoM_{1}=\frac{DRCF}{Power\left(mW\right)}$; $FoM_{2}=\frac{FoM_{1}}{A\left(mm^{2}\right)}$; * Estimated area occupation from die photo.
